# Prognosis in IgA Nephropathy: 30-Year Analysis of 1,012 Patients at a Single Center in Japan

**DOI:** 10.1371/journal.pone.0091756

**Published:** 2014-03-21

**Authors:** Takahito Moriyama, Kayu Tanaka, Chihiro Iwasaki, Yasuko Oshima, Ayami Ochi, Hiroshi Kataoka, Mitsuyo Itabashi, Takashi Takei, Keiko Uchida, Kosaku Nitta

**Affiliations:** Department of Medicine, Kidney Center, Tokyo Women's Medical University, Tokyo, Japan; Institut national de la santé et de la recherche médicale (INSERM), France

## Abstract

**Background:**

Little is known about the long-term prognosis of patients with IgA nephropathy (IgAN).

**Methods:**

This retrospective cohort analysis evaluated clinical and histological findings at the time of renal biopsy, initial treatment, patient outcomes over 30 years, and risk factors associated with progression in 1,012 patients diagnosed with IgAN at our center since 1974.

**Results:**

Of the 1,012 patients, 40.5% were male. Mean patient age was 33±12 years and mean blood pressure was 122±17/75±13 mmHg. Mean serum creatinine concentration was 0.89±0.42 mg/dL, and mean estimated glomerular filtration rate (eGFR) was 78.5±26.2 ml/min/1.73 m^2^. Mean proteinuria was 1.19±1.61 g/day, and mean urinary red blood cells were 36.6±35.3/high-powered field. Histologically, mesangial hypercellularity was present in 47.6% of patients, endothelial hypercellularity in 44.3%, segmental sclerosis in 74.6%, and tubular atrophy/interstitial fibrosis in 28.8% by Oxford classification. Initial treatment consisted of corticosteroids in 26.9% of patients, renin-angiotensin-aldosterone system inhibitor in 28.9%, and tonsillectomy plus steroids in 11.7%. The 10-, 20-, and 30-year renal survival rates were 84.3, 66.6, and 50.3%, respectively. Tonsillectomy plus steroids dramatically improved renal outcome. Cox multivariate regression analysis showed that higher proteinuria, lower eGFR, and higher uric acid at the time of renal biopsy were independent risk factors for the development of end stage renal disease (ESRD).

**Conclusions:**

IgAN is not a benign disease, with about 50% of patients progressing to ESRD within 30 years despite treatment.

## Introduction

Mesangial proliferative glomerulonephritis with mesangial deposition of immunoglobulin A (IgA) and IgG, now recognized as IgA nephropathy (IgAN) was first described in 1968 [1]. Since then, the mechanism of onset and progression of this disease have been described, along with treatment modalities, patient prognosis, and risk factors. Although IgAN was initially regarded as a benign disease, a study in the 1990s reported that about 40% of patients with IgAN progressed to end stage renal disease (ESRD) within 20 years [2], [3]. Since the first report, in 1986, showing that steroid therapy had a beneficial effect in patients with IgAN [4], steroids have been frequently used to treat IgAN patients with massive proteinuria (>1 g/day), active histological findings such as crescent formation, and normal renal function. A randomized controlled trial in 1999 found that steroid pulse therapy could decrease amount of proteinuria and improve patient prognosis [5]. Moreover, steroid pulse therapy was found to have long term efficacy in these patients [6] and has been widely recognized as the most effective treatment for IgAN. Since a retrospective analysis in 2001 found that the combination of tonsillectomy plus steroid pulse therapy induced complete remission of proteinuria and hematuria and prevented deterioration of renal function [7], the number of institutions in Japan performing tonsillectomy plus steroid pulse therapy in patients with IgAN has dramatically increased [8], [9]. Although these intensive therapies may improve the prognosis of patients with IgAN, the mechanisms underlying the onset and progression of IgAN, the risk factors for progression and the long term prognosis of these patients have not been determined. Indeed, despite intensive treatment, many patients with IgAN progress to ESRD.

This retrospective cohort study was performed to analyze the long term outcomes in patients with IgAN and their prognosis over 30 years. We therefore analyzed clinical and histological findings at renal biopsy and risk factors for progression in 1,012 IgAN patients diagnosed at our institution.

## Materials and Methods

### Ethics statement

This cohort study was conducted in accordance with the Declaration of Helsinki, and approved by the Medical Ethics Committee of Tokyo Women's Medical University (#2962). Written informed consent for renal biopsy was obtained from all patients and for use of clinical data at the time of renal biopsy and subsequent histological data was obtained from all recent patients.

### Patients

Between 1974 and 2011, 1,012 patients at Tokyo Women's Medical University were diagnosed with primary IgAN by renal biopsy. IgAN was diagnosed based on light microscopic findings of mesangial proliferative changes, immunofluorescence findings of mesangial IgA and C3 deposition, and electron microscopic findings of electron-dense deposits in the mesangial area. Patients were observed for a mean 7.9±7.1 years (maximum 36 years); 4 patients died during the observation period.

### Histological evaluation of renal biopsy specimens

All renal biopsy specimens were obtained by percutaneous needle biopsy. The specimens were fixed in 10% phosphate-buffered formalin (pH 7.2), embedded in paraffin, and cut into 4-µm-thick sections. The sections were stained with hematoxylin and eosin, periodic acid–Schiff, silver methenamine, and Masson trichrome and examined by light microscopy. The percentage of glomerular lesions, such as global sclerosis, segmental sclerosis or adhesion, cellular or fibrocellular crescents, and fibrous crescents, were evaluated. The histological findings were also graded according to the Oxford classification [11], [12], which scored four key pathological features in each specimen. (i) Mesangial hypercellularity was scored as M0 if >50% of glomeruli had fewer than three cells per mesangial area or M1 if >50% of glomeruli had more than three cells per mesangial area. (ii) Segmental glomerulosclerosis was scored as absent (S0) or present (S1). (iii) Endocapillary hypercellularity was scored as absent (E0) or present (E1). (iv) Tubular atrophy/interstitial fibrosis score was based on the ratio of tubular atrophy/interstitial fibrosis in the total interstitium and scored as T0 (0–25%), T1 (26–50%), or T2 (>51%). Biopsies containing fewer than 8 glomeruli were excluded from analysis.

### Demographic and clinical data

Patient sex, age, body mass index (BMI), systolic blood pressure (S-BP), diastolic blood pressure (D-BP), mean arterial pressure (MAP), and time from onset of IgAN were recorded. In Japan, a large proportion of the population can receive free annual medical examinations, including annual urinary screening. These screenings are regularly performed in school, the workplace, and in municipal facilities. These screenings enable recognition of the onset of IgAN as the first manifestation of a urinary abnormality or a finding of macrohematuria. Laboratory data included serum total protein, albumin, creatinine, uric acid, total cholesterol, HDL-cholesterol, triglyceride, IgG, IgA, IgM, C3, and C4 concentrations; blood urea nitrogen concentration; estimated glomerular filtration rate (eGFR), CH50 activity; urinary protein excretion (U-Prot), and urinary red blood cells (U-RBC). These clinical and laboratory findings were measured at the time of renal biopsy. Time to progression to ESRD, defined as requiring dialysis or renal transplantation, and the risk factors associated with progression to ESRD were also evaluated. Initial treatment, defined as treatment performed during the first year after renal biopsy, and renal survival rate relative to treatment were evaluated. The eGFR was calculated by the modified isotope dilution mass spectorometry – modification of diet in renal disease (IDMS-MDRD) study for Japanese [eGFR = 194×S-Cre^−1.094^×age^−0.287^×0.739 (if female)] [10].

### Statistical analysis

Data are presented as means ± standard deviation (SD) and were analyzed using JMP 10.0.1 (SAS Institute, Cary, NC, USA). Cumulative renal survival rates until ESRD were calculated according to the Kaplan–Meier method and compared using the log rank test. Univariate and multivariate Cox regression analysis were used to evaluate the risk of deterioration to ESRD. In univariate analyses, sex (male/female) was regarded as a categorical variable, whereas age, BMI, MAP, interval from onset, eGFR, serum albumin, uric acid, total cholesterol, U-Prot, U-RBC, IgA, IgA/C3 and histological grading were regarded as quantitative variables. Multivariate analysis included the factors differing significantly in univariate analysis. The results of these univariate and multivariate analyses are expressed as hazard ratios (HRs) with 95% confidence intervals (CIs). In all analyses, *P*<0.05 was considered statistically significant.

## Results

### Clinical findings at the time of renal biopsy

Table 1 shows the clinical characteristics at the time of renal biopsy. Of the 1,012 patients, 40.5% were male. Mean patient age was 32.9±12.0 years, with 41% aged 20–29 years and 25% aged 30–39 years. Mean S-BP was 121.7±17.1 mmHg and mean D-BP was 74.6±12.8 mmHg. Mean MAP was 90.4±12.8 mmHg and 79% of IgAN patients had a MAP below 100 mmHg. Mean interval from IgAN onset to renal biopsy was 7.9±7.1 years. We found that renal biopsies were obtained from 32% of patients within 2 years of IgAN onset and from 21% 10 years after onset.

**Table 1:** Clinical and laboratory findings at renal biopsy.

Variables	Values
***Clinical Findings***	
**Sex**	410/602 (Male/Female)
**Age**	32.9±12.0 years
**Body Mass Index**	21.6±3.1 kg/m^2^
**Systolic Blood Pressure**	121.7±17.1 mmHg
**Diastolic Blood Pressure**	74.6±12.8 mmHg
**Mean arterial Pressure**	90.4±12.8 mmHg
**Interval from onset**	5.8±6.6 years
**Observation period**	7.9±7.1 years
***Laboratory Findings***	
**Total Protein**	6.69±0.73 g/dl
**Serum Albumin**	3.92±0.53 g/dl
**Blood Urea Nitrogen**	15.8±5.7 mg/dl
**Serum Creatinine**	0.89±0.42 mg/dl
**eGFR**	78.5±26.2 ml/min/1.73m^2^
**Uric Acid**	5.71±1.62 mg/dl
**Total Cholesterol**	197.3±48.5 mg/dl
**HDL Cholesterol**	56.4±16.7 mg/dl
**Triglyceride**	123.7±82.2 mg/dl
**IgG**	1158.1±341.2 mg/dl
**IgA**	325.1±126.6 mg/dl
**IgM**	144.7±75.7 mg/dl
**CH50**	40.6±8.4 mg/dl
**C3**	87.5±35.2 mg/dl
**C4**	29.0±15.5 mg/dl
**Urinary protein excretion**	1.19±1.61 g/day
**U-RBC**	36.6±35.3 counts/HF

Table 1 also shows laboratory data at the time of renal biopsy. Mean total protein concentration was 6.69±0.73 g/dl, and mean serum albumin concentration was 3.92±0.53 g/dl. Of these patients, 6% had serum albumin concentrations <3.0 g/dl and 12% had serum albumin concentrations between 3.0 and 3.5 g/dl, with the remainder having serum albumin concentrations over 3.5 g/dl. The mean eGFR was 78.5±26.2 ml/min/1.73m^2^, with 46% of patients having eGFR over 80 ml/min/1.73m^2^, 30% between 60 and 80 ml/min/1.73m^2^, 19% between 40 and 60 ml/min/1.73m^2^, and 5% between 20 and 40 ml/min/1.73m^2^. Mean serum total cholesterol and triglyceride concentrations were 197.3±48.5 mg/dl and 123.7±82.2 mg/dl, respectively. Mean IgA concentration was 325.1±126.6 mg/dl, with 28% of patients having IgA concentrations between 300 and 400 mg/dl, and 23% >400 mg/dl. Mean U-Prot was 1.19±1.61 g/day, with 62% of patients having less than 1 g/day, 30% between 1 and 3 g/day, and 9% over 3 g/day. Mean U-RBC was 36.6±35.3 counts/high powered field (HPF), with 51% of patients having <25/HPF, 20% having between 25 and 50 counts/HPF, 4% having between 50 and 100 counts/HPF, and 20% having >100 counts/HPF.

### Histological findings

Table 2 shows the histological findings in samples taken from patients with IgAN. The mean number of glomeruli per renal biopsy was 16.1±10.4. Over 8 glomeruli were present in biopsy specimens from 858 patients and these samples were used to evaluate glomerular lesions and Oxford classification. Global sclerosis was observed in a mean 15.5±16.6% of samples, segmental sclerosis or adhesion in 13.6±13.7%, cellular or fibrocellular crescents in 6.72±10.5%, and a fibrous crescent in 2.25±5.76%. According to the Oxford classification, 47.6% of samples were classified as M1, 44.3% as E1, 74.6% as S1, 23.0% as T1 and 5.8% as T2.

**Table 2:** Histological findings (glomerular lesions and Oxford classification).

*Glomerular lesions*	Values
** Number of glomeruli**	16.1±10.4 (number)
** Global Sclerosis**	15.5±16.6 %
** Segmental Sclerosis or Adhesion**	13.6±13.7 %
**Crescent**	9.12±12.3 %
** Cellular or Fibro-Cellular**	6.72±10.5 %
** Fibrous**	2.25±5.76 %

*Oxford Classification [number/(%)]*	Values [number(%)]
**Mesangial hypercellularity: M0/M1**	449/408 (52.4/47.6%)
**Endocapillary hypercellularity: E0/E1**	477/380 (55.7/44.3%)
**Segmental glomerulosclerosis: S0/S1**	218/639 (25.4/74.6%)
**Tubular atrophy/Interstitial fibrosis: T0/T1/T2**	610/197/50 (71.2/23.0/5.8%)
**Not evaluated**	155 (15.3%)

### Initial treatment

Table 3 shows the initial treatment of patients with IgAN. We found that 272 patients (26.9%) received corticosteroids; 119 (11.7%) received corticosteroid therapy combined with tonsillectomy; 15 (1.5%) underwent tonsillectomy alone; 15 (1.5%) received immunosuppressive agents such as cyclophosphamide, mizoribine, and rituximab; 293 (28.9%) received a renin-angiotensin-aldosterone system inhibitor; 600 (59.3%) received an antiplatelet/anticoagulation agent; 159 (15.7%) received eicosapentaenoic acid; 3 (0.3%) underwent plasma exchange or double filtration plasmapheresis; and 169 (16.7%) received no treatment.

**Table 3:** Initial treatment of individual patients.

Treatment	Values [number (%)]
Corticosteroid	272 (26.9%)
Steroid combined with tonsillectomy	119 (11.7%)
Tonsillectomy without steroid	15 (1.5%)
Steroid with Immunosuppressing agents	15 (1.5%)
RAS inhibitors	293 (28.9%)
Anti-platelet agents/anti coagulation	600 (59.3%)
EPA	159 (15.7%)
PE/DFPP	3 (0.3%)
No therapy	154 (15.2%)
Unknown	60 (5.9%)

RAS, renin angiotensin system; EPA, eicosapentaenoic acid; PE, plasma exchange; DFPP, double filtration plasmapheresis.

### Long term outcome of IgA nephropathy

**Figure 1:**
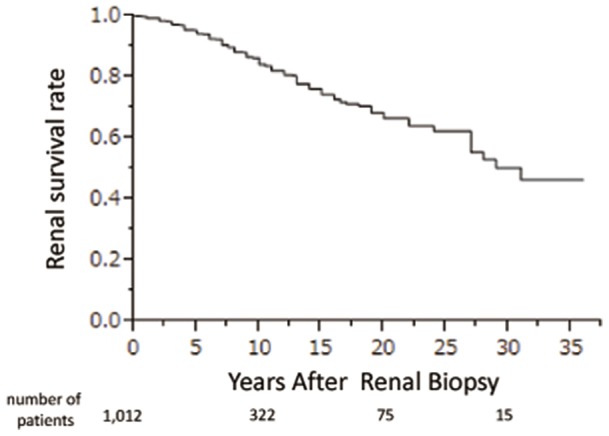
Cumulative renal survival rate from renal biopsy until ESRD in all 1,012 patients with IgAN.

The cumulative 10-, 20- and 30-year renal survival rates, from renal biopsy to ESRD, were 84.3%, 66.6%, and 50.3%, respectively. The 36-year renal survival rate was 46.4% (Figure 1).

### Outcome according to initial treatment

**Figure 2:**
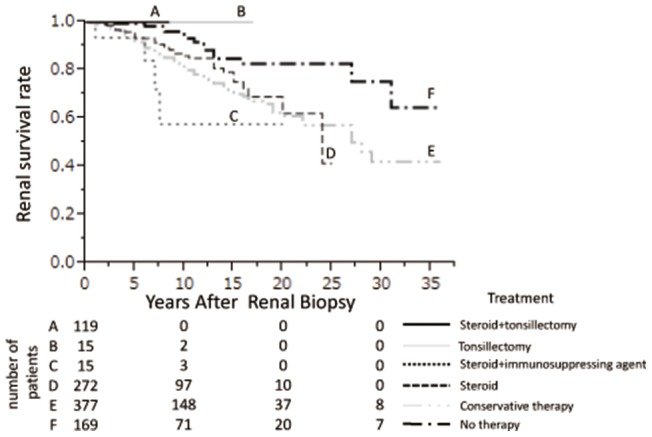
Cumulative renal survival rates in IgAN patients categorized by initial treatment.

The cumulative renal survival rate relative to initial treatment is shown in Figure 2. The 8.5-year cumulative survival rate in patients treated with steroids plus tonsillectomy was 100%, the 17-year survival rate in patients treated with tonsillectomy was 100%, the 20-year survival rate in patients treated with steroids plus an immunosuppressive agent was 57.6%, and the 25-year survival rate in patients treated with steroids was 41.4%. The 35-year renal survival rates in patients treated with conservative therapy (RAS inhibitors and/or anti-platelet agents/anti-coagulation therapy, and/or EPA) and in untreated patients were 42.0% and 64.5%, respectively. The difference among groups was statistically significant (P = 0.0011).

### Univariate and multivariate analysis of factors associated with ESRD

**Figure 3:**
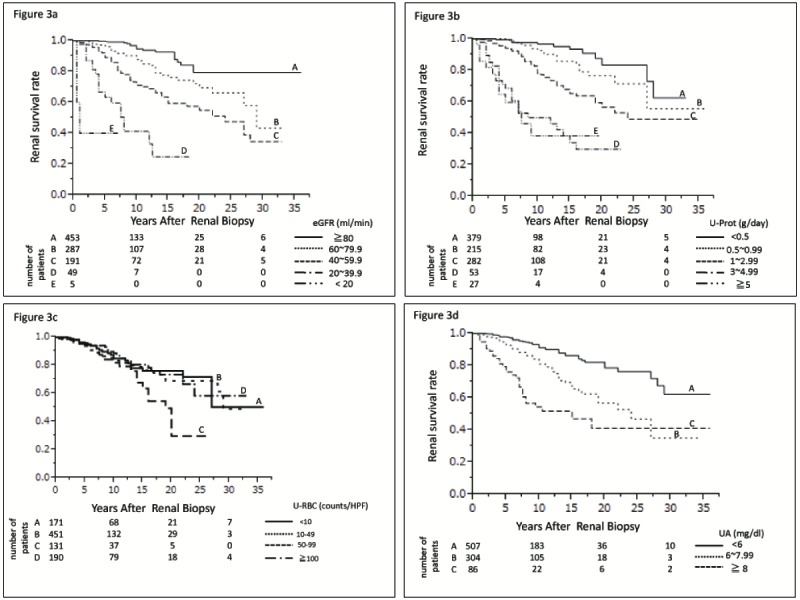
Cumulative renal survival rates in IgAN patients categorized by eGFR, U-Prot, U-RBC and uric acid concentration. (a) The 30-year survival rates of patients with eGFR >80 ml/min/1.73m^2^, between 80 and 60 ml/min/1.73m^2^, and between 60 and 40 ml/min/1.73m^2^ were 80.1%, 43.1%, and 34.6%, respectively. The survival rates of patients with eGFR between 40 and 20 ml/min/1.73m^2^ and <20 ml/min/1.73m^2^ were 24.8% over 18 years and 40.0% over 7 years, respectively. The difference among groups was statistically significant (P<0.0001). (b) The 30-year renal survival rates of patients with U-Prot <0.5 g/day, between 0.5 and 1 g/day, and between 1 and 3 g were 62.6%, 55.6%, and 49.0%, respectively. The survival rates of patients with U-Prot between 3 and 5 g/day and >5 g/day were 29.9% over 20 years and 34.4% over 15 years, respectively. The difference among groups was statistically significant (P<0.0001). (c) The 30-year renal survival rates of patients with U-RBC <10 counts/HF, between 10 and 49 counts/HF, and ≥100 counts/HF were 50.2, 49.0, and 41.7%. The survival rates of patients with U-RBC between 50 and 99 counts/HF was 29.6% over 25 years. The difference among groups was not statistically significant (P = 0.1122). (d) The 30-year renal survival rates of patients with uric acid <6 mg/dl, between 6 and 8 mg/dl, and >8 mg/dl were 62.3, 35.1, and 41.2%, respectively (P<0.0001).

**Figure 4:**
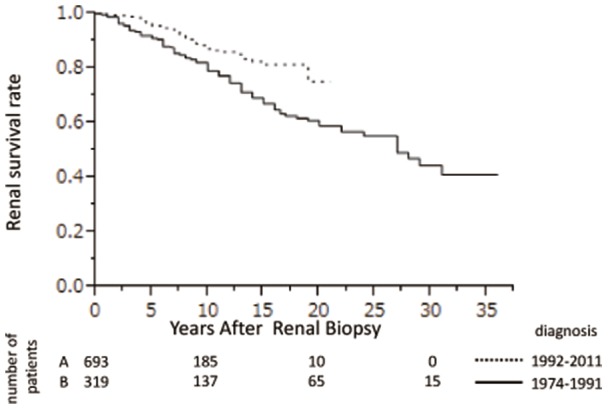
Renal survival rates in IgAN patients diagnosed between 1974 and 1991 and between 1992 and 2001. The 10(86.6% vs. 79.1%) and 20 year (75.2% vs. 59.0%) renal survival rates were significantly higher in patients diagnosed between 1992 and 2011 than in between 1974 and 1991 (P = 0.0002).

Univariate analysis found that male gender, higher age, higher BMI, higher MAP, lower eGFR, lower serum albumin, higher uric acid, higher total cholesterol, higher U-Prot, higher IgA/C3, and higher T level on the Oxford classification were associated with a risk of progression to ESRD. Multivariate Cox regression analysis showed that lower eGFR (HR, 1.93; 95% CI, 1.47–2.56; P<0.0001), higher uric acid concentration (HR, 1.24; 95% CI, 1.054–1.48, P = 0.0176), and higher U-Prot (HR, 1.34; 95% CI 1.07–1.69, P = 0.0116) were independent factors predicting progression to ESRD (Table 4). Kaplan–Meier analysis showed that cumulative renal survival rates were significantly higher in patients with higher than lower eGFR (Figure 3a), in patients with lower than higher U-Prot (Figure 3b), and in patients with lower than higher uric acid concentration (Figure 3d; P<0.0001 each). Cumulative survival, however, did not differ significantly according to the degree of hematuria (Figure 3c, P = 0.1122). Kaplan–Meier analysis also showed that the cumulative renal survival rate was significantly higher in patients diagnosed with IgAN over the last 20 years than during the previous 20 years (75.2% vs. 59.0 %, P = 0.0002; Figure 4).

**Table 4:** Univariate and multivariate analysis of risk factors associated with ESRD in patients diagnosed with IgAN.

	HR	95% CI	P value
***Univariate Analysis***			
male (vs. female)	1.76	1.25–2.49	0.0013
Age (10 year increase)	1.25	1.09–1.44	0.0031
BMI (1 kg/m^2^ increase)	1.10	1.04–1.16	0.0012
MAP (10 mmHg increase)	1.44	1.27–1.65	<0.0001
Interval from onset (1 year increase)	1.02	0.99–1.05	0.0763
eGFR (20ml/min decrease)	2.22	1.86–2.67	<0.0001
Serum Albumin (1 g/dl decrease)	1.99	1.71–2.30	<0.0001
Uric Acid (1 mg/dl increase)	1.57	1.39–1.77	<0.0001
T-Cho (30 mg/dl increase)	1.44	1.26–1.64	<0.0001
U-Prot (1g/day increase)	1.69	1.53–1.86	<0.0001
U-RBC (20/HPF increase)	1.02	0.92–1.13	0.6851
IgA (100 mg/dl increase)	1.09	0.93–1.27	0.2833
IgA/C3 (1 increase)	1.16	1.00–1.35	0.0460
Oxford classification M1(vs. M0)	1.35	0.94–1.97	0.1039
Oxford classification E1 (vs. E0)	1.30	0.89–1.91	0.1733
Oxford classification S1 (vs. S0)	1.23	0.81–1.92	0.3455
Oxford classification T (1 increase)	2.38	1.91–3.20	<0.0001
***Multivariate Analysis***			
male (vs. female)	1.16	0.72–1.90	0.5390
Age (10 year increase)	0.84	0.67–1.03	0.0992
BMI (1 kg/m^2^ increase)	0.93	0.85–1.01	0.0781
MAP (10 mmHg increase)	1.14	0.94–1.38	0.1755
eGFR (20ml/min decrease)	1.93	1.47–2.56	<0.0001
Alb (1 g/dl decrease)	1.24	0.92–1.66	0.1627
Uric Acid (1 mg/dl increase)	1.24	1.04–1.48	0.0176
T-Cho (30 mg/dl increase)	1.17	0.96–1.42	0.1105
U-Prot (1g/day increase)	1.34	1.07–1.69	0.0116
IgA/C3 (1 increase)	1.17	0.96–1.42	0.1111
Oxford classification T (1 increase)	1.06	0.75–1.50	0.7318

HR, hazard ratio; CI, confidence interval; BMI, body mass index; MAP, mean arterial pressure; eGFR, estimated glomerular filtration rate; T-Cho, total cholesterol; U-Prot, urinary protein excretion; U-RBC, urinary red blood cells; IgA, immunoglobulin A.

## Discussion

IgAN is the most common type of chronic glomerulonephritis worldwide [13]. Although slowly progressive over decades, IgAN is not a benign disease. Since IgAN was first described over 40 years ago, patients with this disease have been treated with oral steroids [4], [14], [15], steroid pulse therapy [5], [6], tonsillectomy [7], [16], [17], renin-angiotensin aldosterone system inhibitors [18]–[20], eicosapentaenoic acid [21], statins [22] and combinations of these agents [23]. Although these agents were found to improve short and intermediate term renal outcomes, less is known about very long term outcomes in these patients. In this retrospective analysis, we showed that the 10-, 20-, 30-, and 36-year cumulative renal survival rates were 84.3%, 66.6%, 50.3% and 46.4%, respectively, findings similar to those reported previously for up to 20 years. For example, a study of 282 patients in France showed that the cumulative 10- and 20-year renal survival rates from biopsy to S-Cre >1.5 mg/dl were 84% and 64%, respectively [2]. A study of 502 patients in Japan reported that the 10- and 20-year cumulative survival rates from onset of IgAN until ESRD were 85% and 61%, respectively [3], and a study of 1,155 patients in China found that the 10- and 20-year cumulative renal survival rates until eGFR <15 ml/min/1.73m^2^ were 83% and 64%, respectively [24]. In addition, a study of 1,364 patients in Korea reported that the cumulative 10- and 20-year survival rates from renal biopsy until ESRD or death were 79.8% and 66.9%, respectively [25]. These findings, however, suggested that treatment may not improve prognosis in patients with IgAN because renal survival rates had not increased dramatically over time. We therefore compared renal survival rates in patients diagnosed with IgAN between 1974 and 1991 and those diagnosed between 1992 and 2011, finding that the 20-year survival rate was significantly higher in the latter than in the former group (75.2% vs. 59.0%, P = 0.0002). Similar findings were reported previously [23], confirming that treatment of IgAN improved renal outcomes. Beginning in 2004, patients in our institution have been treated with a combination of steroids with tonsillectomy, with none of the 119 patients treated since 2004 progressing to ESRD. This result, indicating that steroid therapy combined with tonsillectomy improves renal outcomes dramatically, was similar to previous findings [7]. In contrast, 169 patients (16.7% of all patients) received no treatment at the time of renal biopsy, because their clinical and histological findings were mild. These patients had a mean eGFR of 80.9±24.7 ml/min/1.73m^2^ and a mean U-Prot of 0.60±0.69 g/day, with 43.3% classified as M1, 23.3% as E1, 54.1% as S1, 15.8% as T1, and 3.3% as T2. Interestingly, their renal survival rate was better than that of the patients who received steroids, immunosuppressive agents, or conservative therapy as initial treatment, although 35.6% progressed to ESRD in 35 years. These results indicate that, despite their mild clinical and histological findings at the time of renal biopsy, some patients with IgAN progressed to ESRD. Furthermore, careful observation of all IgAN patients is needed, regardless of findings at the time of renal biopsy. Moreover, in our cohort, median (interquartile range) U-Prot (0.68 (0.3–1.305) vs. 0.77 (0.225–1.55) g/day, P = 0.0037) and U-RBC (20 (10–50) vs. 30 (10–100) counts/HF, P<0.0001) were significantly lower and eGFR (80.3 (61.7–99.3) vs. 70.9 (58.5–88.3) ml/min/1.73m^2^, P<0.0001) was significantly higher in patients diagnosed between 1992 and 2011 than in those diagnosed between 1974 and 1991. These findings indicated that more recently diagnosed patients had milder disease at the time of renal biopsy and a better overall prognosis.

In our cohort, about 60% of patients diagnosed as IgAN were female, which contrasts with worldwide findings. We suspected that this may have been due to the name of our institution, Tokyo *Women's* Medical University, which may have attracted more female than male patients. More women than men were found to undergo underwent renal biopsy [13], with several reports from Japan yielding results similar to ours [8], [23], [31]. In addition, two reports from Asia, evaluating over 1,000 IgAN patients, found that the gender distribution was even [24], [25]. The frequency of IgAN in Japan was similar to that of other nations in East Asia, but different from that of other regions in the world [13]. These results indicated that specific genetic and ethnic backgrounds may be related to gender distribution of this disease. Using the Oxford classification, we found that 47.6% of patients could be classified as M1, 44.3% as E1, 74.6% as S1, 23.0% as T1, and 5.8% as T2, although including criteria different from those of the original Oxford classification. We selected all patients diagnosed with IgAN in our institution, including patients with U-Prot <0.5 g/day or eGFR <30 ml/min/1.74^2^, patient not selected by the original Oxford classification. Except for M1, however, the distribution of patients in our cohort was similar to that of the cohort from the original Oxford classification, in which 81% of patients were classified as M1, 42% as E1, 76% as S1, 30% as T1, and 5% as T2 [11], [12]. The lower percentage of our patients classified as M1 may have been due to the large percentage of patients with mild IgAN, defined as U-Prot <0.5 g/day.

Multivariate Cox regression analysis showed that risk factors associated with deterioration to ESRD were impaired renal function and severe proteinuria at the time of renal biopsy, findings similar to those of previous reports [2], [3], [23], [24], [25]. Kaplan–Meier analysis also indicated that renal function and proteinuria affected renal outcome. Acute deterioration of renal function after 25 years was observed in patients with very low proteinuria (<0.5 g/day). The renal survival rate, which was 83.4% at 25 years after renal biopsy, decreased to 73.0% at 27 years and 62.6% at 28 years. However, only nine patients were available for follow-up after 25 years in patients with low proteinuria, with two of these nine patients progressing to ESRD. One of these two patients had diabetic nephropathy and the other had multiple organ failure with severe infection, suggesting that, in the absence of these complications, their renal function may not have deteriorated after 25 years. Other risk factors reported associated with ESRD included uncontrolled hypertension [3], [23], [24], [25] and severe histological damage [3], [23], [25], although none of these was significant in our analysis. We also found that age, sex, hematuria, IgA/C3 ratio and serum albumin were not associated with deterioration to ESRD. Kaplan–Meier analysis also indicated that hematuria did not affect renal outcome. Interestingly, uric acid concentration was a risk factor for progression to ESRD by multivariate Cox regression analysis and Kaplan–Meier analysis. Indeed, higher uric acid concentration has been associated with glomerular damage [26], [27], tubulointerstitial change [26], [27], [28], and vascular damage [26], as well as with poor prognosis [24], [26], [27], [29], and it was independent from renal function [24], [27], further suggesting the importance of controlling uric acid concentration in patients with IgAN.

Univariate analysis showed that the only Oxford classification associated with patient prognosis was T grade (HR 2.38, 95% CI 1.91–3.20, P<0.0001), whereas M, E, and S grades were not. Following multivariate analysis, however, T grade was no longer prognostic, whereas clinical factors, such as lower eGFR, higher U-Prot, and higher uric acid were powerful predictors of renal deterioration. Similar findings have been observed in several other studies. For example, Kaplan–Meier analysis showed no significant differences in renal survival rate in patients assorted by M, E, and S grades, with T grade being the only Oxford classification predictive of renal outcome [30]. Another study reported that higher T grade and extracapillary lesions were risk factors for progression to ESRD, as were lower eGFR, higher U-Prot and the absence of steroid treatment, whereas M, E, and S grades were not [31]. Multivariate analysis showed that the most powerful predictor of renal outcome was renal function at biopsy, with eGFR, U-Prot and hypertension being prognostic; in contrast, none of the Oxford classifications was predictive of progression to ESRD [32]. Although Oxford classification is recognized as useful in predicting renal outcomes, these findings suggest that the Oxford classification be evaluated in prospective, large sized, worldwide validation studies.

Our study had several limitations, including the relatively low number of patients followed-up. Of the 1,012 patients who underwent of renal biopsy, only about 30% could be evaluated at 10 years, and only 6% at 20 years. Loss of patients with milder disease to follow-up, with more severely ill patients remaining under observation, may have biased our results towards more severe outcomes. In conclusion, we assessed long term renal survival in patients with IgAN, as well as evaluating factors associated with renal survival. We found that about 50% of patients with IgAN developed ESRD within 30 years. Higher U-Prot, lower eGFR, and higher uric acid were independent risk factors for the development of ESRD.
